# Exploring cued and non-cued motor imagery interventions in people with multiple sclerosis: a randomised feasibility trial and reliability study

**DOI:** 10.1186/s40945-018-0045-0

**Published:** 2018-03-02

**Authors:** Barbara Seebacher, Raija Kuisma, Angela Glynn, Thomas Berger

**Affiliations:** 1 School of Health Sciences, University of Brighton, Robert Dodd Building, 49 Darley Road, Eastbourne, BN20 7UR UK; 2 Clinical Department of Neurology, Medical University of Innsbruck, Anichstrasse 35, 6020 Innsbruck, Austria

**Keywords:** Multiple sclerosis, Physiotherapy, Motor imagery, Rhythmic cueing, Walking, Fatigue, Quality of life, Motor imagery ability, Feasibility, Reliability two-dimensional gait analysis

## Abstract

**Background:**

Motor imagery (MI) is increasingly used in neurorehabilitation to facilitate motor performance. Our previous study results demonstrated significantly improved walking after rhythmic-cued MI in people with multiple sclerosis (pwMS). The present feasibility study was aimed to obtain preliminary information of changes in walking, fatigue, quality of life (QoL) and MI ability following cued and non-cued MI in pwMS. The study further investigated the feasibility of a larger study and examined the reliability of a two-dimensional gait analysis system.

**Methods:**

At the MS-Clinic, Department of Neurology, Medical University of Innsbruck, Austria, 15 adult pwMS (1.5–4.5 on the Expanded Disability Status Scale, 13 females) were randomised to one of three groups: 24 sessions of 17 min of MI with music and verbal cueing (MVMI), with music alone (MMI), or non-cued (MI). Descriptive statistics were reported for all outcomes. Primary outcomes were walking speed (Timed 25-Foot Walk) and walking distance (6-Minute Walk Test). Secondary outcomes were recruitment rate, retention, adherence, acceptability, adverse events, MI ability (Kinaesthetic and Visual Imagery Questionnaire, Time-Dependent MI test), fatigue (Modified Fatigue Impact Scale) and QoL (Multiple Sclerosis Impact Scale-29). The reliability of a gait analysis system used to assess gait synchronisation with music beat was tested.

**Results:**

Participants showed adequate MI abilities. Post-intervention, improvements in walking speed, walking distance, fatigue, QoL and MI ability were observed in all groups. Success of the feasibility criteria was demonstrated by recruitment and retention rates of 8.6% (95% confidence interval, CI 5.2, 13.8%) and 100% (95% CI 76.4, 100%), which exceeded the target rates of 5.7% and 80%. Additionally, the 83% (95% CI 0.42, 0.99) adherence rate surpassed the 67% target rate. Intra-rater reliability analysis of the gait measurement instruments demonstrated excellent Intra-Class Correlation coefficients for step length of 0.978 (95% CI 0.973, 0.982) and step time of 0.880 (95% CI 0.855, 0.902).

**Conclusion:**

Results from our study suggest that cued and non-cued MI are valuable interventions in pwMS who were able to imagine movements. A larger study appears feasible, however, substantial improvements to the methods are required such as stratified randomisation using a computer-generated sequence and blinding of the assessors.

**Trial registration:**

ISRCTN ISRCTN92351899. Registered 10 December 2015.

**Electronic supplementary material:**

The online version of this article (10.1186/s40945-018-0045-0) contains supplementary material, which is available to authorized users.

## Background

### Introduction

Multiple Sclerosis (MS) is a chronic disease of the central nervous system, which leads to destruction of the protective myelin nerve sheaths and accumulating disability. People with MS have impairment in motor, sensory, visual and other body systems [1]. Evidence shows that 40–80% of patients report fatigue [2, 3] which, together with walking impairment, contributes to a limitation in their walking endurance [4] and independence in daily life activities [5]. Therefore, it is considered essential to develop novel rehabilitation strategies to improve walking and fatigue, which negatively impact walking abilities.

### Motor imagery

Apart from physical training, motor imagery (MI) has increasingly been used in neurorehabilitation to enhance motor relearning in people with neurological disorders [6]. Defined as mental rehearsal of a movement, the motor action is not executed [7, 8]. Motor areas of the brain responsible for movements’ physical execution are activated, although to a lesser degree, during imagined movements [6–9]. Both real and imagined movements are associated with similar motor preparation processes [7, 8]. In addition, imagined and actual movements have previously been shown to share similar temporal profiles [8, 10]. Mental chronometry, that is temporal congruence, is the similar time duration of imagined and real motor actions [10, 11]. Consequently, the capacity to preserve the temporal features of a movement during MI has been associated with a person’s MI ability [11, 12]. Mental chronometry studies showed that the MI accuracy and its temporal organisation were impaired in participants with MS versus controls; these deficits in MI ability were associated with cognitive impairment, but were independent from motor functioning [13–16]. Reduced MI accuracy and timing are also related to depression [14]. However, rhythmic auditory cueing has been found to promote the MI ability in people with MS, by optimising their MI duration and movement amplitudes during an upper limb task [17].

### Rationale

To our knowledge, only one study explored the effects of rhythmic-cued MI in 15 people with stroke [18]. Kim et al. observed an improvement in walking performance after kinaesthetic rhythmic-cued MI practice, compared to visual MI practice without auditory cueing [18]. Similarly, results from our previous study showed significant improvements in walking performance after four weeks of music- and metronome-cued kinaesthetic MI practice with additional verbal cueing in people with MS and mild to moderate disability [19]. Music-cued MI was more effective than metronome-cued MI in improving fatigue and quality of life (QoL). However, we did not measure the participants’ ability to imagine movements or their actual gait synchronisation with music beat. We also did not include a non-cued MI group and thus could not compare between the effects of cued and non-cued MI. In other words, there are various areas of uncertainty in knowledge concerning the mechanisms of the rhythmic-cueing and MI interventions in persons with MS, which have not yet been addressed. For example, the contributions from the MI practice and the music and verbal cueing, respectively, to the functional improvements have not been investigated. To our knowledge, only one study explored the effect of five weeks of MI practice on fatigue, walking speed and QoL in 20 people with MS and mild to moderate disability [20]. Their findings showed that fatigue and QoL significantly improved, but the walking speed improvement was not significant.

Rhythmic auditory stimulation has been successfully used to improve walking performance [21]. Hence, we hypothesised that rhythmic auditory cueing of the MI would serve as an external timekeeper to the imagined steps. Thereby, like in real walking [22], imagery of walking along a regular and hence predictable auditory rhythm might be processed quite accurately, mainly because the imagined steps are produced slightly ahead of the cue [21]. This coupling process between external rhythm and body adjustment is referred to as rhythmic entrainment which, for example, causes the synchronisation of gait with the tempo of music beat [21, 22]. Against the background of these findings, we suggested that rhythmic cueing might induce entrainment and enhance the MI ability in our participants. To the best of our knowledge so far, no study has evaluated the mechanisms of differently cued and non-cued MI for walking rehabilitation in people with MS. Thus, we plan to conduct a randomised controlled trial (RCT) to investigate the effects and mechanisms of differently cued and non-cued MI on walking, fatigue, QoL, MI ability and gait synchronisation with music beat in people with MS. The present feasibility study was used to gather preliminary results and examine the feasibility of the aforesaid RCT.

### Aims

The aims of this feasibility study were:

1. to explore the success of the feasibility criteria: recruitment and retention rates and adherence rate;
2. to explore the safety of the interventions, adverse events and participant acceptability of the interventions;
3. to obtain preliminary information on change in walking speed and walking distance induced by the two types of music-cued and non-cued MI;
4. to acquire preliminary information on change in fatigue produced by the two types of music-cued and non-cued MI;
5. to collect preliminary data on the change in QoL generated by the two types of music-cued and non-cued MI;
6. to evaluate the baseline MI ability and obtain preliminary information on its change;
7. to assess the reliability and repeatability of the quantitative gait measurement instruments used to assess synchronisation with music beat.

## Methods

### Study design and location

A three-group parallel randomised controlled single-centre feasibility trial was conducted. The adjunct CONSORT checklist for pilot and feasibility studies is available as Additional file 1 [23]. All measurements were taken at the MS Clinic of the Clinical Department of Neurology, Medical University of Innsbruck, Austria.

### Participants and recruitment

**Fig. 1:**
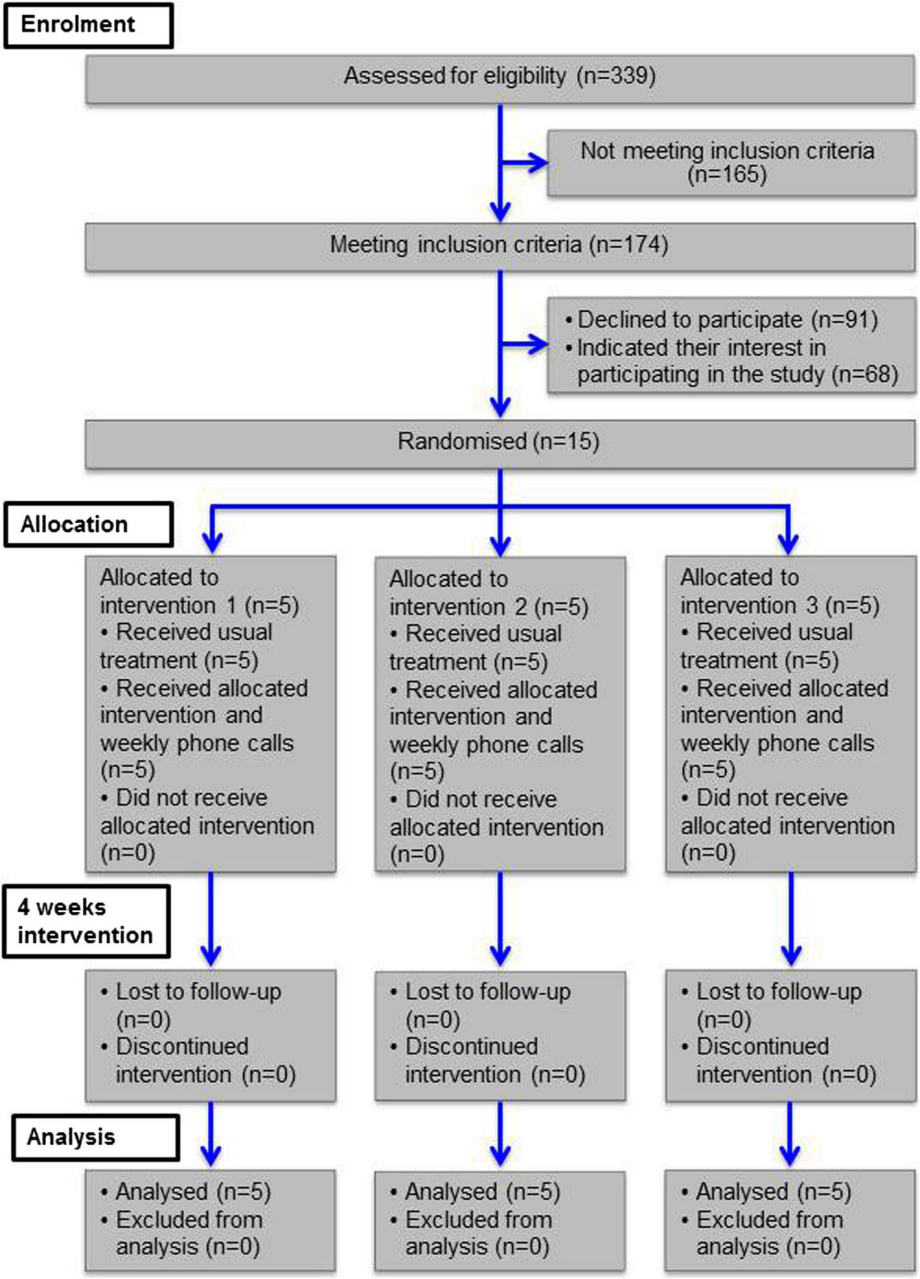
CONSORT flow diagram for pilot and feasibility studies

Recruitment used unselected consecutive sampling and restricted randomisation from 3rd March to 14th April 2016. Due to the piloting character of this study and time constraints, 15 participants were recruited into three groups. The MS-Clinic currently cares for approximately 2500 people with MS, 339 were screened during recruitment, of those 174 were eligible. A CONSORT flow diagram for pilot and feasibility studies [23] is shown in Fig. 1.

Inclusion Criteria were: people with any MS phenotype according to McDonald’s criteria [24], mild to moderate disability (Expanded Disability Status Scale, EDSS 1.5 to 4.5) [25], aged 18 years or over, any ethnicity, German speaking (questionnaires, MI familiarisation, instructions).

Exclusion Criteria were: concomitant diseases which may affect rhythmic cued MI or walking (e.g. orthopaedic disorders, untreated hearing loss), a relapse of MS within the last three months, recent start of physiotherapy treatment or change of medication which is known to affect walking within the last two months, known pregnancy, overt symptoms or signs of depression or cognitive dysfunction diagnosed and documented by the Innsbruck MS Clinic. A relapse during the intervention period would have led to the exclusion from the study.

Eligible individuals were identified during their usual visit to the Outpatient MS Clinic by their neurologist (TB). After that, they received the recruitment flyer from the reception staff. They also obtained the participant information sheet and detailed verbal information on the study from the researcher who is a physiotherapist (BS). Oral and written information was presented in comprehensible lay language. Eligible individuals had the opportunity to ask questions. They had at least twenty-four hours to consider their participation and to consult family members or others. After one week, potential participants who agreed to telephone contact, were called by the researcher asking if they would like to participate in the study. Written informed consent was obtained by the researcher. Participants were then randomly allocated using a 1:1:1 ratio, and they were asked to draw a sealed envelope, prepared by a researcher not involved in this study, with numbers ´1′, ´2′ or ´3′ which corresponded with the three study groups. The randomisation was restricted insofar as only 15 envelopes were provided. Using this procedure, the physiotherapist involved in the recruitment could not influence group allocation.

### Intervention

The intervention of this study consisted of music- and verbally-cued MI (MVMI group), music-cued MI (MMI group) and non-cued MI (MI group). After the randomisation and prior to the intervention, study participants were familiarised with rhythmic-cued MI or non-cued MI by the researcher (BS), as suggested in previous studies [26, 27]. These authors have proposed the PETTLEP approach to MI in neurorehabilitation, involving the “Physical, Environmental, Task, Timing, Learning, Emotional, and Perspective” components of MI [28]. The PETTLEP checklist is based on neuroscientific findings and has been developed by Holmes and Collins (2001, page 60) for performance improvements in athletes. The PETTLEP elements concern the physical, or bodily, position of the practitioner including arousal, the imagined environment, the imagined task, the MI timing, the learning or changes induced by the MI, the emotions or affective states, which are related to the MI task, and the MI perspective. The PETTLEP ideas were applied to the context of the present study to enhance the effectiveness of the intervention.

Firstly, participants were informed about the concept of (cued) MI and its application in sports and neurorehabilitation. Secondly, participants also learned about the different *perspectives* (internal and external) and modes (visual, kinaesthetic) which can be adopted during MI [29, 30]. In the visual mode, the persons imagine *watching* themselves (internal perspective) or other people (external perspective) moving, and in the kinaesthetic mode, the persons *experience or feel* themselves moving [31]. When employed in clinical practice, people use both perspectives when asked to imagine a movement. Therefore, participants had the opportunity to try them out for themselves and become aware of their preferred mode or perspective. The researcher placed emphasis on internal, kinaesthetic MI, which was adopted for the intervention and MI assessment of this study and the internal, visual MI which was only used for the MI ability testing. During the training, participants were asked for MI content characteristics such as the mode and perspective they were using, for the *environment* or for movement aspects they were imagining (*learning*). Moreover, the duration of the actual and imagined walking performance was compared to monitor the mental process [26]. Participants were asked to walk a six-metre distance along the marked hallway while the time was measured. After that, they were asked to imagine themselves walking the same six-metre distance they had reached and indicate when they imagined reaching the same point. The time was measured, and feedback was given to the participants. If desired, they could repeat the imaginative task as many times as they wanted. The intervention is presented in Table 1 and was based on the Template for Intervention Description and Replication (TIDieR template) [32].

**Table 1:** Intervention chart

ITEM NO	ITEM DESCRIPTION		
1 BRIEF NAME	MVMI Group	MMI Group	MI Group
	Music- and verbally cued MI	Music-cued MI	Non-cued MI
2 WHY	PETTLEP approach to MI (Holmes and Collins 2001)		
3 WHAT MATERIALS	- Study CDs or dropbox link including the audio mix and download to smartphone, laptop, tablet or MP3-player		
	- 4 sessions on each CD, one for each week		
	- Headphones or earphones could be used if desired		
Study CD Content	- Kinaesthetic MI instructions	- Kinaesthetic MI instructions	- Kinaesthetic MI instructions
	- Instrumental music in 2/4 or 4/4 m	- Instrumental music in 2/4 or 4/4 m	
	- Emphasis of every first beat, or every first and third beat by rhythmic verbal cues (e.g. “toe-off” or “step-step”)		
	For example, music titles used in week two were: Unheilig, Der Berg (Intro), 82 bpm; Black, Wonderful Life, 106 bpm; Malcolm Arnold, The River Kwai March, 120 bpm; Uriah Heep, Lady in black, 86 bpm; Abba, Dancing queen, 101 bpm; Toto, Africa, 100 bpm; DJ Bobo, I’m living to love you, 110 bpm; Katy Perry, Firework, 120 bpm.		No cueing
Availability of CDs	After completion of the main study, a download of the 3 study CDs will be available upon request from the corresponding author.		
4 WHAT PROCEDURES	- MI introduction, familiarisation and training: in lay language; description of the concept of MI; its application and effects in sports and neurorehabilitation; principles of neuroplasticity; MI perspectives (internal and external) and modes (visual, kinaesthetic).		
	- Measurement of actual and imagined walking duration over a 6-m distance to monitor the mental process		
	- Performance feedback for participants and repeated training if desired		
	- Weekly phone calls for support and adherence reports		
	- Additional introduction to rhythmic auditory stimulation plus its use in neurorehabilitation		
	- Rhythmic-cued MI familiarisation		
PETTLEP Elements			
Position (Physical)	- Practise at any time of the day when alert		
	- Seated in an upright body position		
	- Shoulders relaxed		
	- Avoid tightening the muscles or moving		
	- Eyes closed		
	- Normal breathing		
Environment	- Practice in a quiet place at home		
	- Imagine walking indoors (long hallway similar to that in the MS Clinic) and walking outdoors (on a straight and familiar path)		
Tasks	- Take long strides		
	- Take giant strides		
	- Roll your feet on the ground and feel your body weight on your soles		
	- Touch the ground with your heels first		
	- Raise the front of your feet		
	- Raise your knees		
	- Pace		
	- Place/feel your weight on your feet		
	- Place/feel your weight on your legs		
	- Stamp your feet while walking		
	- Walk effortlessly, almost as if you were floating		
	- Walk forcefully and energetically as if you were an athlete		
	- March as if you were in the army		
	- Walk in an extremely upright posture such as when balancing a sachet, filled with rice, on your head		
	- Feel the swinging of your arms while walking		
	- Feel the swinging of your legs while walking		
Timing	External timing was provided: “imagine yourself walking in time with the music and verbal cues”	External timing was provided: “imagine yourself walking in time with the music”	Timing was internal and depended on the tempo and intensity of the walking tasks.
	- Tempo (cadence) was between 80 and 120 steps/min		
	- Slow, medium and fast music pieces alternated, with a general progression in the tempo		
	- The imagined walking tempo was consistent with the music beat at 80–120 bpm.		
Learning	- See familiarisation		
	- Additionally, weekly phone call support was provided		
Emotion	MI instructions included motivational and arousal enhancing aspects (e.g. walk forcefully and energetically as if you were an athlete). See instructions under Tasks.		
	Motivational instrumental music was used with the MI		
Perspective	Kinaesthetic MI from an internal, first-person perspective		
5 WHO PROVIDED	The intervention including the preparation of the CDs was provided by the researcher (BS), an experienced physiotherapist with 11 years of musical training.		
6 HOW	- MI introduction, familiarisation and training: individually or in small groups (2–3 participants) and depending on the group they were allocated to		
	- Monitoring of mental process: individually		
	- Weekly phone calls: individually		
7 WHERE	- MI introduction, familiarisation, training and monitoring of mental process: at MS Clinic Innsbruck, Department of Physiotherapy		
	- Cued MI practice: At participants’ homes		MI practice: At participants’ homes
8 WHEN AND HOW MUCH	17 min, 6 times a week, for 4 weeks		
9 TAILORING	Same intervention for all participants	Same intervention for all participants	Same intervention for all participants
10 MODIFICATIONS	No modifications	No modifications	No modifications
11 HOW WELL PLANNED	- Intervention adherence was assessed using a participant diary and also during weekly phone calls and at post-intervention		
	- Recording in excel sheets was performed by the researcher (physiotherapist) who instructed participants		
12 HOW WELL ACTUAL	The adherence rate was median 5 (range 4, 6) times per week or 83% (95% confidence interval 0.42, 0.99).		

Abbreviations: MI: motor imagery; bpm: beats per minute

Selection of the music style, beat patterns and tempo were based on relevant literature in the field [21]. Music beat in the MVMI and MMI groups was in 2/4 or 4/4 m and in addition, in the MVMI group, every first beat, or every first and third beat were stressed and emphasised by rhythmic verbal cues [21, 22]. Appropriate rhythmical sequences were cut and mixed (GarageBand, Apple Inc.) with the MI instructions of walking. Karaoke music pieces were selected from a wide range of musical styles, including rock, pop, folk music, dance, techno and marching music, hard rock and film music.

Participants were asked to practice MI six times a week, once a day for seventeen minutes over a four week period. After each week and in all groups, the audio mix was changed to facilitate adherence and to retain attention with the MI [21], so that four mixes, designed in the same way, were on one CD. Participants were called weekly to support them with the MI and as a reminder of the practice. The phone calls were made by the researcher, who introduced the participants to the (cued) MI practice and explained all the procedures. Questions that were asked during the phone calls are added as Additional file 2.

### Data collection

Demographic (gender, age) and MS disease specific data (current EDSS) were extracted from patients’ charts, study specific assessment data were collected pre and post intervention by one physiotherapist who is not a member of the MS Clinic. The German version of all assessments was used. Baseline and post-intervention assessments were performed at the physiotherapy department of the MS Clinic, always in the mid-afternoon, in view of daytime fluctuations in walking abilities and fatigue. Participants were allowed to rest at any time during the instructions and assessments. A history of depression and cognitive dysfunction before onset or its worsening or new occurrence after diagnosis of MS was rigorously asked and documented by the same treating neurologist (TB). Clinical definitions of depression and cognitive dysfunction were used rather than formal neuropsychological testing. Depression was defined as a state of low mood and loss of activity along with characteristic symptoms such as sadness, anxiety, awkwardness, loss of appetite, insomnia, up to suicidal thoughts. Cognitive dysfunction was defined by report and clinical assessment of characteristic symptoms such as impairment in orientation, memory, attention, learning, language, visuospatial skills, calculating, planning or any other executive function.

### Primary outcomes

#### Feasibility

Feasibility of conducting a full-scale RCT was evaluated. The criteria for feasibility success were: a) a target recruitment rate of 5.7% out of 174 eligible patients (or 10 participants per month), b) a target retention rate of 80% and c) a target minimum adherence rate of 67% (4 practice sessions per week out of a maximum of 6).

#### Safety, adverse events and acceptability

Participants were asked to report any adverse event such as falls, excessive fatigue, psychological distress and/or other safety related occurrences. Severe adverse events would have led to early study termination. During the weekly phone calls, participants were asked for their feedback on the study procedures, which were recorded in Excel files and reported. Participant adherence with the interventions was noted by participants in a diary. Adherence and the acceptability of the interventions were reported narratively. Acceptability referred to kinaesthetic MI, melodies, beat tempo and the verbal cueing. Questions that were asked by the researcher are available as additional information; see Additional file 2.

### Secondary outcomes

#### Walking speed and walking distance

Walking speed was assessed by the Timed 25-Foot Walk (T25FW) [33]. The T25FW is the most commonly reported short walking test, which has excellent validity, reliability and responsiveness in people with MS [34] and was administered according to instructions in the Multiple Sclerosis Functional Composite [35]. There is a consensus in the literature that a change of 20% and above in walking speed corresponds to a clinically meaningful change, or minimal clinically important difference (MCID), in walking [36].

Walking distance was measured by the 6-Minute Walk Test (6MWT) [37]. The 6MWT was carried out as recommended by the American Thoracic Society-Guidelines. Learmonth et al. (2013) demonstrated that a change of 20% and above in walking distance represents the minimal detectable change (MDC) [38], reflecting the smallest real difference which exceeds the measurement error [39, 40]. Other research reported that a change in walking distance of 20% and above is clinically meaningful [41]. Based on clinical judgement, a 20% change in walking distance is relevant for the patients in their daily lives. Good to excellent psychometric properties of the 6MWT have been demonstrated in an MS population [37]. 6MWT reference values for healthy women and men aged 20 to 50 are mean (standard deviation, SD) 593 ± 57 m and 638 ± 44 m, respectively, with differences depending on height and age [42]. Females and males with MS and an EDSS below 4.0 walked mean (SD) 380.1 ± 156.0 m and 459.5 ± 133.8 m, respectively; women and men with an EDSS of up to 6.5 walked 322.2 ± 156.4 m and 362.8 ± 169.2 m, with variations connected to age, cardiorespiratory function and balance [43].

Walking aids for both the T25FW and 6MWT were used if required and were documented and kept consistent during the two assessments.

### Fatigue

Fatigue was assessed using the Modified Fatigue Impact Scale (MFIS). The MFIS is a modified version of the Fatigue Impact Scale [44] and one part of the Multiple Sclerosis Quality of Life Inventory (MSQLI) [45]. It is a 21 item Likert scale that evaluates, via self-report, the effects of fatigue on physical, cognitive and psychosocial functioning, with higher numbers indicating greater fatigue. All items can be answered by five categories (never, rarely, sometimes, often and almost always; range 0–4) resulting in a total score from 0 to 84. The MFIS has an excellent reliability and moderate to high validity and responsiveness in people with MS [46, 47]. Based on previous studies, the cut-off value for MS-related fatigue was set at ≥38 points on the MFIS total score [48]. Responsiveness of the MFIS, as expressed by the smallest detectable change (SDC) is − 16.2 points on the total score, − 8.9 points on the physical subscale, − 8.0 points on the cognitive subscale and − 2.3 points on the psychosocial subscale [47].

### Quality of life

QoL was assessed with the Multiple Sclerosis Impact Scale (MSIS-29). The MSIS-29 is a 29-item disease-specific, patient-reported questionnaire for measuring the impact of MS on individual lives, with 20 items associated with a physical subscale and 9 items with a psychological subscale [49]. Responses use a 5-point Likert scale, ranging from (1) “not at all” to (5) “extremely”. The MSIS-29 showed the strongest psychometric properties compared to other QoL scales [50, 51]. The items ask about the impact of MS on day-to-day living over the past two weeks, higher scores indicating a greater impact of MS on daily function. According to a Rasch analysis, the two scales are different and should not be combined to a total score [52]; in the current study, the analysis was performed accordingly.

### Motor imagery ability

Evidence has recommended to comprehensively assess the MI ability using at least two different approaches because some people may have problems generating vivid images and intense sensations and/or subjectively assessing their MI ability, and others with the duration of their mental imagery, in relation to real movements [53, 54]. In other words, both questionnaire and mental chronometry (that is, temporal congruence) tests are required to assess the MI capability and both were used in this study.

The Kinaesthetic and Visual Imagery Questionnaire (KVIQ-10) is the short version of the KVIQ-20, which is a MI ability questionnaire developed for people with physical disabilities [55, 56]. Thus, the questionnaire is researcher-administered, and the movements are easily performed while participants are seated [55, 56]. The KVIQ-10 consists of a visual (V) and kinaesthetic (K) subscale and assesses the clarity of the image (V) and the intensity of the sensations (K) on a five-point ordinal scale [55]. The five visual categories range from (1) “no image” to (5) “image as clear as seeing”; the five kinaesthetic categories range from (1) “no sensation” to (5) “as intense as executing the action”. The maximum KVIQ-10 total scale is 50 points, with 25 points in each subscale, and higher scores representing higher levels of MI ability. The KVIQ-10 has been attributed excellent validity and reliability in people with stroke [55] and MS [57]. For this study, the validated German version of the KVIQ-10 was used, the KVIQ-G-10 [56]. The minimum expected level of the MI ability at baseline and post-intervention, as assessed by the KVIQ-G-10, was median 3 out of 5 points on both subscales [58].

The Time-Dependent MI (TDMI) screening test is a mental chronometry test which measures the number of imagined stepping movements in seated participants over 3 time periods (15, 25 and 45 s) [53]. Good to excellent reliability of the TDMI was shown in people with stroke with intra-class correlation coefficients (ICCs) between 0.87 and 0.93 [12].To encourage kinaesthetic MI from a first-person perspective, participants were asked to close their eyes and feel themselves moving their right or left leg to a board placed in front of their feet.

### Repeatability and reliability of gait analysis instruments

**Fig. 2:**
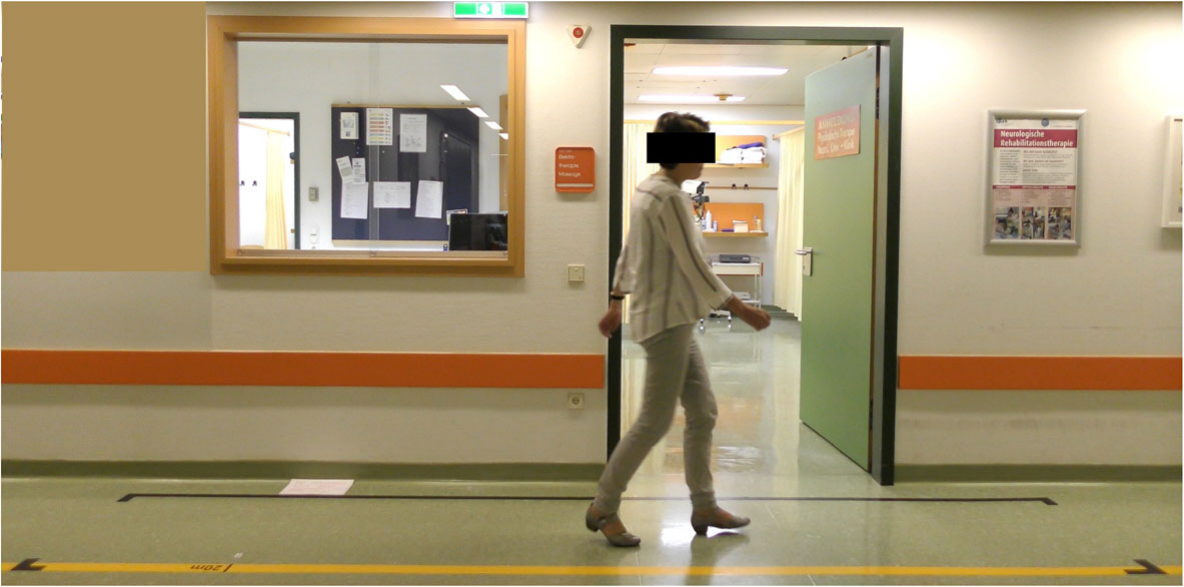
Representative image of the 2D video-based gait assessment

Participants were asked to synchronise their steps with instrumental music in regular metre at 110 bpm. The choice of the music beat frequency of 110 bpm was based on the gait literature in people with MS [59–61]. People with mild to moderate MS showed a mean (SD) cadence of 109.1 ± 23.3 steps/min [59]; patients with MS and higher disability levels walked mean 98.97 ± 19.95 steps/min [60]. The cadence in pwMS with an EDSS of up to 5.7 was between 100.0 ± 23.3 steps/min and 112.1 ± 11.3 steps/min [61]. The music was played with an Apple i-phone and X-Mi X Mini II Capsule-Loudspeakers in a calm hallway free from obstacles. Gait synchronisation analysis with musical pulse requires knowing step lengths and step times, therefore, 2-dimensional (2D) video recording in the frontal plane was performed [62, 63]. The videos were taken with a Panasonic HC-WX979 4 K camcorder with a frame rate of 50 fields per second, which was mounted on a 1.3 m high Haehnel 9,994,180 Triad 40 Lite Tripod placed 5 m from the participant [62, 64]. Participants walked between two marked lines, 1 m apart, on a 30 m hallway, so that they could accelerate and decelerate their speed, and adjust their gait to the music beat. Thus, only the central 4.5 m were video-recorded while the participant walked 4 to 6 times back and forth, depending on their step length (Fig. 2). Participants were allowed to wear shoes or walk barefoot and the type of footwear or its absence was to be kept consistent during all trials. This procedure enabled the acquisition of 25–35 steps per participant. These video footages were used to measure the average step length and step time per participant (test measures). The video-footage was analysed using CCC Utilius Fairplay 5 Software. This software allowed calibration of the field of view, in order to reduce parallax error [64]. Step length was defined as the distance between the initial contact of one foot with the floor, followed by the opposite foot, and was measured in metres [64]. Step time was defined as the period between the initial contact of one foot and the other foot, and was measured in seconds [64]. In other words, the crucial moment was the heel contact with the ground, or, in the case of severe spasticity, the toe contact. Excellent image exposure was achieved, due to the camcorder 4 K technology and a well-lit hallway, therefore, no reflective markers were used.

**Fig. 3:**
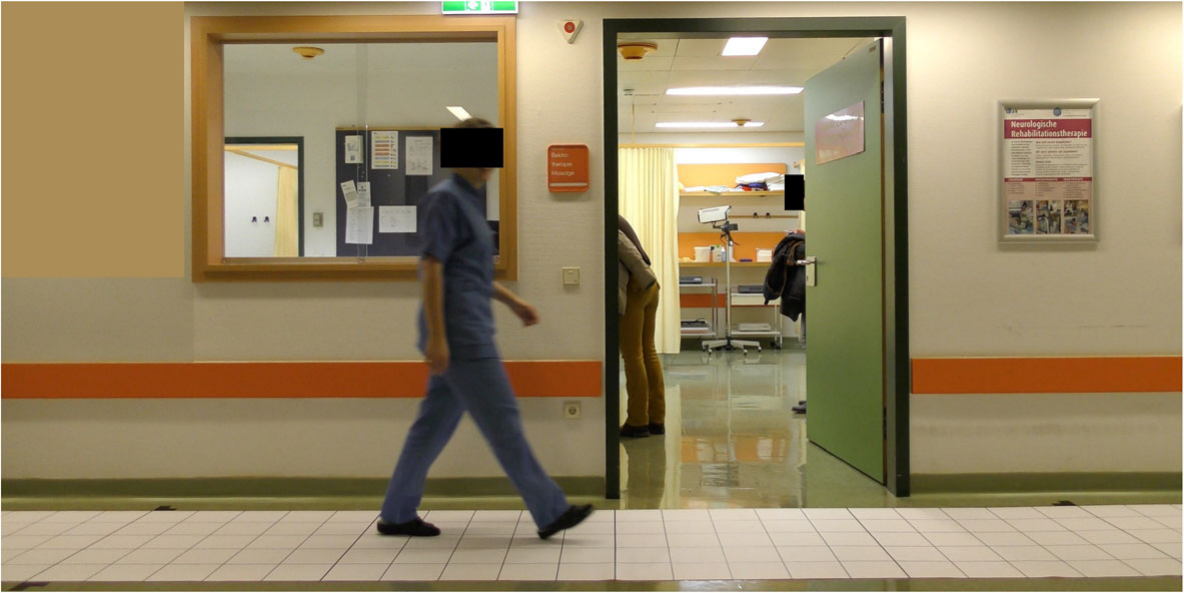
Representative image of the accuracy evaluation of the video analysis software calibration

Prior to the study, the accuracy of the video analysis software calibration grid was evaluated, by using a 7 × 1 metres large grid mat with 0.2 × 0.2 m large grid fields which accurately covered the marked walkway (Fig. 3). Accuracy was confirmed, as the grid fields of the grid mat and the video calibration grid were congruent. Moreover, the measurement technique described above was established in 13 healthy team colleagues. After that, this feasibility study assessed the repeatability and reliability of the gait analysis system. In addition to the test measures described above, the step lengths and step times were measured 4 times on the video footage by the same rater, using Utilius Fairplay 5 software. From each of these measures the mean step length and step time was calculated, so that 4 retest measures were available.

### Statistical analysis

All statistics were performed using IBM SPSS software, release 24.0 (IBM Corporation, Armonk, NY, USA) and GraphPad Prism 6 (GraphPad, San Diego, California, USA). Intention-to-treat analysis was performed for all cases, which were analysed by the originally assigned groups. Descriptive statistics were reported for all outcomes. Medians (interquartile ranges) were reported for continuous and ordinal data: fatigue, QoL, KVIQ-G-10 MI ability (all: 5 categories), TDMI MI ability, walking speed and walking distance; medians (range) were reported for age, compliance (7 categories: 0–6 times per week), and disability (EDSS). Means (95% CI) were reported for reliability data, and raw count (frequency, percentage) for nominal data (recruitment rates, retention rate, missing data, falls and adverse events). Median MI vividness scores were calculated by dividing the median KVIQ-G-10 scores by the number of items, that is, 5 for the visual/kinaesthetic subscales, and 10 for the total score. Spearman’s correlation coefficients (median with interquartile ranges; all baseline pairwise correlations) were calculated for the numbers of imagined stepping movements over 15, 25 and 45 s (TDMI). Differences between the three groups at the baseline were examined using Fisher’s Exact test (nominal data: gender; walking aid use and fatigue yes/no) and Kruskal Wallis test (continuous data: age; walking speed and walking distance; ordinal data: fatigue, QoL, MI ability). Corrected Fisher’s exact contingency table analysis was calculated using an online calculator (http://www.physics.csbsju.edu/stats/exact_NROW_NCOLUMN_form.html).

The recruitment rate (%) was determined by dividing the number of participants who consented by the number of patients eligible, multiplied by 100. The retention rate was estimated: (N who completed the study/N total sample)*100, where N is the number of participants. The adherence rate was reported as the percentage of the scheduled (cued) MI practice (6×/week), actually performed by the participants over the 4 week study period [65]. The eligibility, recruitment and adherence rates were calculated with their 95% CI according to the Wilson ‘score’ method cited by Newcombe [66]; when the proportion was close to 0 or 1, a Poisson approximation as described by Brown was used [67].

The ICC was estimated as a measure of intra-rater reliability. The ICCs were calculated with their 95% CI using a two-way mixed model based on absolute agreement with single measure analysis. Point estimates of the ICC were rated as excellent (0.9–1), good (0.73–0.9), moderate (0.4–0.74) and poor (0–0.39) [68]. The standard error of measurement (SEM) was calculated to assess the random error, or variability, of the step length and step time measurements. It is the difference between an observed score and its ‘true’ score [69]. The SEM was calculated using the formula: SEM = SD*√(1-ICC), where SD is the sample standard deviation [70, 71]. The SEM was multiplied by 1.96 in order to compute the error that is expected in 95% of the measurements [69]. This value was then multiplied by √(2) to compute the MDC_95_, i.e. the smallest amount of change between two repeated measurements, based on a 95% confidence interval, which is beyond the measurement error and hence is a genuine change [72].

## Results

### Baseline characteristics

Participants´ baseline data are shown in Table 2 and Additional file 3. The female-to-male ratio was 6.5:1, as only two males participated in this study. Participants in the MI group were younger and walked faster and a longer distance. In the MI group, one participant used two walking sticks during all walking tests. Participants in the MVMI group were more disabled, showed higher fatigue values, lower QoL and lower kinaesthetic MI ability. These differences between the groups did not reach statistically significance. Apart from slightly lower values in the MVMI group, with a 25th percentile of 2.7, participants seemed to have been able to practice MI, as indicated by median KVIQ-G-10 vividness scores of 4.0 (interquartile range 3.2, 4.6) out of 5.0. Additionally, there was a moderate to strong positive mean Spearman’s correlation of r = 0.78 (interquartile range 0.77, 0.84) for the numbers of imagined stepping movements during 15, 25 and 45 s for both lower limbs.

**Table 2:** Baseline characteristics

Parameter	Group 1	Group 2	Group 3	*P*-value
	Music and verbally cued motor imagery (MVMI)	Music cued motor imagery (MMI)	Motor imagery (MI)	
	*N* = 5	*N* = 5	*N* = 5	
Gender^a^ (F:M)	*N* = 4:1	*N* = 5:0	*N* = 4:1	0.999^d^
Age (years)^b^	52.0 (41.0, 69.0)	54.0 (34.0, 72.0)	37.0 (27.0, 74)	0.500^e^
EDSS^b^	4.5 (2.0–4.5)	2.5 (2.5–4.5)	2.5 (1.5–4.5)	0.387^e^
Walking aid use during testing^a^				
No/unilateral/bilateral aid	N = 5/0/0	*N* = 5/0/0	N = 4/0/1	0.999^d^
T25FW^c^ (seconds)	5.6 (5.2, 6.2)	7.0 (5.3, 7.0)	4.9 (4.6, 5.4)	0.264^e^
6MWT^c^ (metres)	367.5 (348.0, 435.2)	380.6 (337.2, 392.0)	460.5 (442.9, 476.8)	0.249^e^
MFIS physical sub^c^	21.0 (2.0, 25.0)	12.0 (10.5, 25.0)	16.0 (9.0, 21.0)	0.983^e^
MFIS cognitive sub^c^	17.0 (11.5, 22.5)	3.0 (0.0, 16.5)	11.0 (11.0, 14.0)	0.098^e^
MFIS psychosoc sub^c^	2.0 (1.0, 4.0)	2.0 (0.0, 5.0)	3.0 (1.5, 5.0)	0.638^e^
MFIS total score^c^	40.0 (17.5, 51.0)	28.0 (13.0, 39.0)	30.0 (23.5, 38.0)	0.564^e^
MFIS total score ≥ 38	*N* = 3/2	*N* = 2/3	*N* = 1/4	0.800^d^
MSIS-29 physical sub^c^	45.0 (17.5, 49.4)	20.0 (17.5, 39.4)	16.2 (10.6, 35.6)	0.296^e^
MSIS-29 psychological sub^c^	27.8 (18.0, 33.3)	11.1 (17.5, 39.4)	11.1 (8.3, 25.0)	0.137^e^
KVIQ-G-10 visual sub^c^	21.0 (17.5, 23.5)	20.0 (17.5, 24.5)	21.0 (17.5, 24.0)	0.992^e^
Median visual score^c^	4.2 (3.5, 4.7)	4.0 (3.5, 4.9)	4.2 (3.5, 4.8)	0.992^e^
KVIQ-G-10 kinaesthetic sub^c^	15.0 (13.5, 18.5)	24.0 (15.5, 24.5)	23.0 (18.0, 23.5)	0.189^e^
Median kinaesthetic score^c^	3.0 (2.7, 3.7)	4.8 (3.1, 4.9)	4.6 (3.6, 4.7)	0.189^e^
KVIQ-G-10 total^c^	40.0 (31.0, 40.0)	43.0 (33.5, 49.0)	44.0 (36.0, 47.0)	0.328^e^
Median total score^c^	4.0 (3.1, 4.0)	4.3 (3.3, 4.9)	4.4 (3.6, 4.7)	0.328^e^
TDMI 25 s right^c^	15.0 (12.0, 22.0)	14.0 (12.0, 16.0)	16.0 (11.5, 23.0)	0.688^e^
TDMI 15 s left^c^	10.0 (9.0, 11.5)	9.0 (7.5, 11.0)	13.0 (6.5, 14.5)	0.572^e^
TDMI 45 s right^c^	27.0 (19.5, 36.0)	23.0 (19.0, 31.0)	33.0 (20.0, 38.5)	0.664^e^
TDMI 15 s left2^c^	10.0 (8.5, 12.0)	8.0 (8.0, 9.5)	12.0 (7.5, 13.0)	0.231^e^
TDMI 25 s left^c^	16.0 (14.5, 19.5)	16.0 (14.5, 18.0)	20.0 (12.0, 21.0)	0.883^e^
TDMI Spearman’s r^c^	0.72 (0.47, 0.82)	0.59 (0.46, 0.72)	0.87 (0.80, 0.97)	

Abbreviations: N: Counted number of participants; F:M: Females: Males; EDSS: Expanded Disability Status Scale; MFIS: Modified Fatigue Impact Scale; MFIS physical sub: MFIS physical subscale; MFIS cognitive sub: MFIS cognitive subscale; MFIS psychosoc sub: MFIS psychosocial subscale; MSIS-29 physical sub: Multiple Sclerosis Impact Scale-29 physical subscale; MSIS-29 psychological sub: Multiple Sclerosis Impact Scale-29 psychological subscale; KVIQ-G-10: Kinaesthetic and visual imagery questionnaire, German short version; KVIQ-G-10 visual sub: KVIQ-G-10 visual subscale; KVIQ-G-10 kinaesthetic sub: KVIQ-G-10 kinaesthetic subscale; KVIQ-G-10 total: KVIQ-G-10 total score; r: Spearman’s correlation coefficient (all pairwise correlations; all significant ≤0.05); TDMI: Time-dependent motor imagery screening test; T25FW: Timed 25-Foot Walk; 6MWT: 6-Minute Walk Test

^a^Counted number of participants

^b^Median (range)

^c^Median (interquartile range)

^d^Analysed with corrected Fisher’s Exact contingency table analysis

^e^Analysed with Kruskal Wallis test

### Primary outcomes

#### Feasibility

- 174 out of 339 people with MS were eligible for the study, corresponding to an eligibility rate of 51.3% (95% CI 46.0, 56.6%) (see Fig. 1). Of these 174 participants, 15 consented to the study within one month, which is a recruitment rate of 8.6% (95% CI 5.2, 13.8%). This recruitment rate exceeded the target recruitment rate of 5.7%.
- All 15 participants completed the study and there were no missing data, both corresponding to a 100% retention rate (95% CI 76.4, 100%). This retention rate surpassed the target retention rate of 80%.
- With reference to a maximum practice frequency of 6 times per week, participants reported to have practised median 5 (range 4, 6) times per week. This adherence rate of 83% (95% CI 0.42, 0.99) was greater than the target adherence rate of 67%.

#### Safety, adverse events and acceptability

No safety-related events such as falls occurred in this study. No adverse events related to this home-based study were reported. Phone calls were considered supportive by participants. One participant in the MI group reported minor concentration problems during the MI which resolved with practice. Overall, participants in the MI group appeared to be satisfied with the interventions. All participants in the MVMI and MMI groups reported that they liked the music styles, melodies, and tempo changes of the music pieces. Moreover, 9 out of 15 participants reported as an adjunctive comment that they found safe and convenient practicing the intervention at home, in a sitting position. To summarise, the interventions were found to be acceptable or even pleasurable.

### Secondary outcomes

#### Walking speed and walking distance

**Fig. 4:**
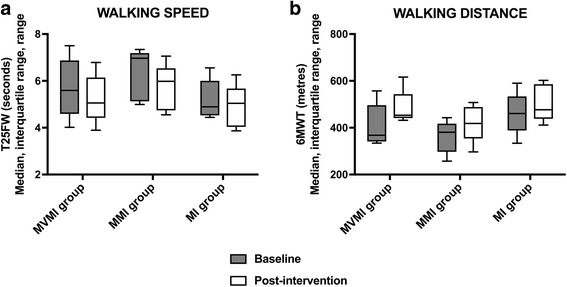
Walking performance pre- and post-intervention. **a** Walking Speed. **b** Walking distance. Medians are shown by lines in the centre of the box-plots; the interquartile ranges are indicated by the boxes and ranges by the whiskers. Abbreviations: MI group = non-cued motor imagery group; MMI group = music-cued motor imagery group; MVMI group = music- and verbally cued motor imagery group; T25FW = Timed 25-Foot Walk; 6MWT = 6-Minute Walk Test. The grey boxes indicate the baseline data and the white boxes present the post-intervention data

Change in walking performance between baseline and post intervention for participants in all groups is presented in Table 3 and Fig. 4. Improvements in walking speed and walking distance were observed in all groups with the greatest walking distance improvements in the cued MI groups. Only one participant in the MVMI group reached a clinically significant improvement in walking speed of ≥20%. Three participants in the MVMI group and two participants in both MMI and MI groups showed a clinically meaningful improvement in walking distance of ≥20% from baseline to post intervention.

**Table 3:** Walking, fatigue and quality of life post-intervention data for each study group

Parameter	MVMI group	MMI group	MI group
T25FW (seconds)			
Change from baseline^a^	−0.1 (−0.7, −0.1)	−0.4 (−0.9, −0.3)	−0.4 (− 0.4, − 0.3)
Post-intervention^a^	5.1 (5.0, 5.5)	6.0 (4.9, 6.0)	5.0 (4.2, 5.1)
Participants with clinically significant improvement (≥20%)	*N* = 1/5	*N* = 0/5	*N* = 0/5
6MWT (metres)			
Change from baseline^a^	85.5 (59.4, 97.1)	65.1 (39.5, 74.8)	33.6 (11.6, 77.7)
Post-intervention^a^	453.2 (450.5, 470.0)	418.0 (412.0, 469.0)	476.5 (465.0, 569.3)
Participants with clinically significant improvement (≥20%)	*N* = 3/5	*N* = 2/5	*N* = 2/5
MFIS physical subscale			
Change from baseline^a^	−3.0 (−7.5, 4.0)	−2.0 (−13.5, −0.5)	−3.0 (−6.5, −1.5)
Post intervention^a^	14.0 (9.0, 19.5)	9.0 (1.0, 21.5)	12.0 (7.5, 15.0)
MFIS cognitive subscale			
Change from baseline^a^	−4.0 (−5.5, 0.5)	−3 (−9.5, 0.0)	−2.0 (−4.5, −0.5)
Post intervention^a^	15.0 (10.0, 18.0)	0.0 (0.0, 7.0)	10.0 (6.5, 13.0)
MFIS psychosocial subscale			
Change from baseline^a^	0.0 (−1.0, 0.0)	0.0 (−3.5, 0.0)	−2.0 (−2.5, −0.5)
Post intervention^a^	2.0 (0.5, 3.5)	0.0 (0.0, 2.5)	1.0 (0.0, 3.5)
MFIS total score			
Change from baseline^a^	−9.0 (−13.0, 4.5)	−6.0 (−23.5, −3.0)	−9.0 (− 10.5, −4.5)
Post intervention^a^	26.0 (22.0, 41.0)	12.0 (2.5, 28.0)	23.0 (15.0, 30.5)
MFIS total score ≥ 38 (post-intervention)	*N* = 2/5	*N* = 0/5	*N* = 0/5
MSIS-29 physical subscale			
Change from baseline^a^	−1.2 (− 11.9, 3.7)	−7.5 (− 13.7, − 2.5)	− 2.5 (−8.1, 6.2)
Post intervention^a^	27.5 (18.7, 48.1)	16.2 (8.1, 30.6)	21.2 (10.6, 30.0)
MSIS-29 psychological subscale			
Change from baseline^a^	0.0 (−11.1, 9.7)	−5.5 (−6.9, 2.8)	−5.5 (−9.7, 1.4)
Post intervention^a^	30.6 (11.1, 37.5)	5.6 (0.0, 23.6)	8.3 (4.2, 19.4)

Abbreviations: *T25FW* Timed 25-Foot Walk, *6MWT* 6-Minute Walk Test, *MFIS* Modified Fatigue Impact Scale, *MFIS physical* MFIS physical subscale, *MFIS cognitive* MFIS cognitive subscale, *MFIS psychosocial* MFIS psychosocial subscale, *MFIS total* MFIS total score, MSIS-29 = MS Impact Scale-29; N: Counted number of participants

With walking speed (T25FW), improvement is indicated by a minus and worsening by a plus; with walking distance (6MWT), improvement is indicated by a plus and worsening by a minus

^a^Median (interquartile range)

### Fatigue

**Fig. 5:**
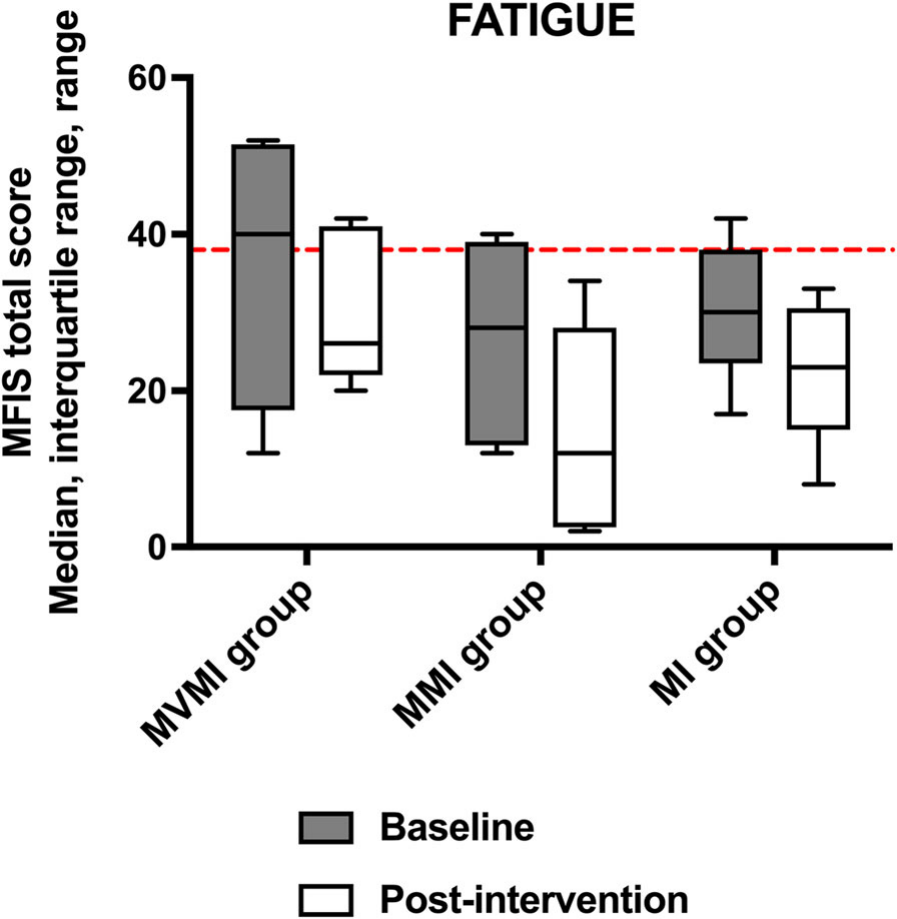
Total fatigue pre- and post-intervention. The red line represents the cut-off point for fatigue as defined at ≥38 points on the MFIS [48]. Medians are shown by lines in the centre of the box-plots; the interquartile ranges are indicated by the boxes and ranges by the whiskers. Abbreviations: MFIS = Modified Fatigue Impact Scale; MI group = non-cued motor imagery group; MMI group = music-cued motor imagery group; MVMI group = music- and verbally cued motor imagery group. The grey boxes indicate the baseline data and the white boxes present the post-intervention data

As can be seen in Table 3 and Fig. 5 and was consistent with our previous results, improvement in fatigue was observed in all groups. At baseline, 6 out of 15 participants in all groups had fatigue, with ≥38 points on the MFIS total score, which was reduced to 2 participants in total from the MVMI group.

### Quality of life

**Fig. 6:**
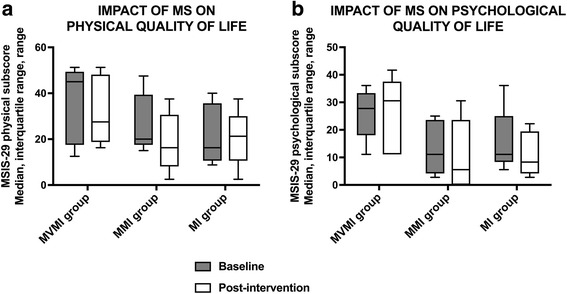
Quality of life pre- and post-intervention. **a** Physical quality of life. **b** Psychological quality of life. Medians are shown by lines in the centre of the box-plots; the interquartile ranges are indicated by the boxes and ranges by the whiskers. Abbreviations: MI group = non-cued motor imagery group; MMI group = music-cued motor imagery group; MSIS-29 = Multiple Sclerosis Impact Scale-29 MVMI group = music- and verbally cued motor imagery group

As shown in Table 3 and Fig. 6, QoL improved in all groups or remained at least stable. Throughout the study, the lowest QoL values were observed in the MVMI group.

### Motor imagery ability

**Fig. 7:**
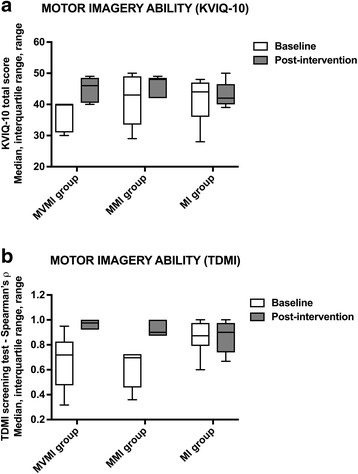
MI ability pre- and post-intervention. **a** MI vividness. **b** Mental chronometry during MI. Abbreviations: KVIQ-G-10 = Kinaesthetic and Visual Imagery Questionnaire-10, German short version: Medians are shown by lines in the centre of the box-plots; the interquartile ranges are indicated by the boxes and ranges by the whiskers; MI group = non-cued motor imagery group; MMI group = music-cued motor imagery group; MVMI group = music- and verbally cued motor imagery group. The grey boxes indicate the baseline data and the white boxes present the post-intervention data

Post-intervention, as compared to baseline, participants showed higher MI abilities. The MI ability, as assessed by median total KVIQ-G-10 scores, reached similar values in all groups, with 4.6 (interquartile range 4.0 to 4.8) points in the MVMI group, when compared to 4.8 (interquartile range 4.2 to 4.8) points in the MMI group and 4.2 (interquartile range 4.0 to 4.6) points in the MI group. Better MI abilities were also suggested by an overall median KVIQ-G-10 score of 4.0 (interquartile range 4.1 to 4.8) and strongly correlated TDMI measures of median r = 0.89 (interquartile range 0.84 to 0.91). Strong correlations of the TDMI measures were observed in all groups, as presented in Fig. 7.

### Reliability of gait analysis instruments

Results from the reliability analysis of the gait analysis system are shown in Table 4. Excellent intra-rater reliability between measures was found, as evidenced by ICCs around 0.98 for step length and of 0.88 for step time. The SEM for repeated measures was 0.013 m and for step time 0.014 s. In other words, the SEM was small to moderate for step length measures and small for step time measures. The MDC_95_ for step length was 0.037 m and for step time 0.038 s. This means that only when an individual’s change from baseline to post-intervention or follow-up in step length and step time exceeds 3.7 cm or 0.038 s one can be 95% confident that the individual was actually changed.

**Table 4:** Repeatability and reliability of gait measurement instruments

Gait parameters	ICC (95% CI)	SEM	MDC_95_
Step length	0.978 (0.973, 0.982)	0.013 m	0.037 m
Step time	0.880 (0.855, 0.902)	0.014 s	0.038 s

Abbreviations: *SEM* standard error of measurement, *ICC (95% CI)* intraclass-correlation coefficient (95% confidence interval), *MDC*_*95*_ minimum detectable change with a 95% confidence interval

## Discussion

As far as we know, this study was the first study to explore the feasibility of the methods to be used for a full-scale RCT that will investigate the mechanisms and effects of cued and non-cued MI on walking, fatigue and QoL in people with MS.

### Feasibility

The observed recruitment, retention and adherence rates exceeded the pre-specified target rates so that a larger study appears feasible. The phone support could have added to adherence rates of median 5 (range 4–6) out of 6. We expect a similar adherence rate in the main trial, mainly because the interventions were acceptable, if not pleasurable to participants. Nevertheless, we recognise that even with careful monitoring via phone-calls, participants could have stated their adherence rates slightly incorrectly.

### Safety, adverse events and acceptability

Participants spontaneously reported that they considered the interventions safe and convenient because they were allowed to practise the MI at home and in a sitting position. As there were no falls or adverse events, it seems safe to continue with the study procedures. 10 out of 10 participants in both music-cued MI groups reported that they liked the music styles and they regarded the intervention acceptable, with 8 out of 10 participants reporting the music intervention as pleasurable. Based on the high acceptability of the music styles, the same music will be used in the main study. All participants in the non-cued MI group were satisfied with the intervention, and two participants viewed the intervention as pleasurable as they appreciated the focus on their body awareness without any distraction. These findings are not unexpected as the melody and beat of music have been shown to impact on emotional function, the urge to move and the perception of fatigue [73]. In addition, we selected appropriate music pieces following participant feedback from our previous study, so as to use a higher amount of music pieces with a faster beat. At first glance, pure MI practise might be less stimulating than music-cued MI, however, the repeated attention towards the MI could positively impact on movement coordination.

We narratively assessed the acceptability of the interventions in this study as we consider high acceptability an extremely important fact; as otherwise, no-one would like to practise nearly every day. In addition, the motivation and commitment during the home-based practice would be lower, which might be accompanied by tiredness, particularly when sitting with closed eyes.

### Walking

The preliminary analysis from this study showed an improvement in walking speed and walking distance after both types of cued MI and non-cued MI. Consistent with our previous results, the greatest improvement in walking distance was seen after music- and verbally-cued MI. We suggest that the regular rhythmic cueing, both by music and verbally, facilitated the brain’s temporal mechanisms similar to that found during cued gait training [22]. Rhythmic-cued MI could then have induced a synchronisation of imagined walking with the beat [21, 22]. Additionally, repeated rehearsal of imagined walking may have facilitated motor learning in all groups [54]. Moreover, as demonstrated in the sports domain and physiotherapy practice, motor tasks need to be specifically trained, in order to produce relevant improvements [74]. The same may apply to MI training, given the comparable characteristics of executed and imagined movements and an overlapping brain area activation during real movement and MI [75]; hence in the current project, a specific training of imagined walking was used. Finally, based on the walking improvements we speculate that cued and non-cued MI rehearsal induced neural plasticity, which could be an underlying mechanism [76], however, this was outside the scope of this study.

### Fatigue

Post intervention, our preliminary results showed a mild improvement in fatigue in all groups when compared to baseline. These findings are in line with results from Catalan et al. (2011) who found significantly improved fatigue after non-cued MI practice in people with MS also at 6 month follow-up [20]. These results also agree with our previous study in people with mild to moderate MS where we observed an improvement in fatigue after rhythmic-cued MI [19].

### Quality of life

To evaluate the impact of the two types of music-cued MI and non-cued MI on the quality of the participants’ day-to-day lives, this study also obtained preliminary information on MS-related QoL. Improvements in physical QoL were observed in all groups; surprisingly and inconsistently with our previous findings, psychological QoL improved after music-cued and non-cued MI, but remained stable after music- and verbally-cued MI. These results seem to contradict the acceptability reports of participants, and probably could be associated with the lowest baseline QoL in participants of the MVMI group and their higher disability, as evidenced by median EDSS scores of 4.5, when compared to 2.5 in the other groups; however, these between-group differences were not statistically significant.

### Motor imagery ability

In accordance with the literature about the multifaceted construct of MI, we used two different approaches to assess the ability to imagine movements in participants, an MI questionnaire and a mental chronometry test [9, 12]. MI ability testing showed that all participants seemed to have been able to practice MI as indicated by median KVIQ-G-10 values of 4.0 (interquartile range 3.2, 4.6) out of 5.0. These scores imply adequate MI vividness [55, 58] and are consistent with a study from Heremans et al. (2012) in 30 people with MS who observed very similar KVIQ-10 scores [13]. However, in the absence of statistically significant group differences, participants in the MVMI group had lower baseline kinaesthetic MI scores than the other groups. The discrepancy in results could be associated with their higher disability and fatigue. Post-intervention, the greatest improvement in the kinaesthetic MI ability was observed in the MVMI group, so that their scores were comparable to the MMI and MI groups. These improvements could be associated with motor learning; motor performance curves typically start with a slowly ascending slope, followed by a sharp increase in performance improvement over a period of practice [77]. In agreement with another study from Heremans et al. (2012), who found that external cueing significantly improved the MI quality in people with MS, we suggest that the music- and verbal cueing enhanced their kinaesthetic MI capability [17]. Starting from higher baseline MI capabilities in the MMI group, a smaller improvement was seen after the intervention. A larger study is required to explore underlying mechanisms.

On the TDMI screening test, the numbers of imagined stepping movements of the left and right lower extremities were strongly related to the three different time periods of 15, 25 and 45 s, as evidenced by Spearman’s correlations of median 0.78 (interquartile range 0.77, 0.84) to the three different time periods of 15, 25 and 45 s. These results are suggestive of high temporal congruence of imagined stepping movements [12]. Our results appear to contradict findings from previous studies that used different mental chronometry tests, related to the upper extremities [13–15]. However, these authors linked impaired MI in this population to cognitive dysfunction [13, 15] and depression [14]. Therefore, we did not include persons with cognitive impairment and/or depression in our study. However, we did not use formal testing for cognitive function and mood, by which we could have obtained accurate results. Importantly, we do not intend to withhold the treatment from people with MS and cognitive impairment or depression in the main study, but it does not seem useful to recruit patients into an MI intervention group when the literature has already demonstrated their diminished ability to perform MI.

### Reliability and repeatability

Evaluation of the 2D quantitative gait analysis system showed excellent reliability and repeatability. This is a substantial requirement to assess the degree of gait synchronisation with music beat in the main study. Our results are in line with work from Harris-Hayes et al. (2014) who examined the reliability and validity of a 2D video based quantitative gait analysis system, and found substantial to excellent reliability between three raters, in addition to excellent validity [62]. Similarly, Norris and Olson (2011) analysed concurrent validity and reliability of 2D video-based motion analysis of the knee and hip joint movement [63]. Using an appropriate motion analysis software and goniometer measures for joint angles, they found excellent concurrent validity. Their reliability measures showed excellent intra- and inter-rater as well as intra-rater reliability. Recently, Paul et al. (2016) assessed the validity and reliability of a 2D motion analysis instrument. They compared an established and sophisticated 3D motion analysis system against a 2D motion analysis system including a commercially available camera, both with the corresponding video analysis software [78]. The authors found excellent agreement between the 2D and 3D systems during balance testing, and excellent reliability of the 2D system.

In this study, we did not use reflective markers to better identify specific body parts, because we were mainly interested in measuring initial contact or heel-strike points of the gait cycle. Thanks to modern camcorder 4 K technology and a well-lit hallway, excellent image exposure was achieved, so reflective markers were redundant. Our suggestions were supported by recent work from Castelli et al. [79] who compared a 2D motion analysis in the sagittal plane with and without markers [79]. They observed high correlations between kinematic measures across different gait speeds for all joints between the two techniques.

The results from our reliability study showed that the gait analysis system was a reliable instrument with low measurement errors related to step time data (SEM 0.014 s) and low to moderate measurement errors related to the step length data (SEM 0.013 m). Accordingly, the MDC_95_ for the step time measures was 0.038 s and 0.037 m for the step length measures. Comparable research showed similar measurement errors for step time (SEM 0.001–0.009 s) and step length (SEM 0.03–0.07 m) measures while using a 2D video-based gait analysis in healthy people [80]. In agreement with this, a further study, which investigated the SEM and MDC using an electronic walkway, found measurement errors for step time of 0.007 s in younger individuals and of 0.015 s in older people, respectively; the SEM for step length was 0.006 m and 0.017 m in the same population. The same study found a MDC of 0.019 s and 0.042 s for step time and of 0.016 m and 0.047 m for step length for younger and older people, respectively [81]. Consistent with this, the above cited study from Paul et al. found measurement errors of 0.024–0.064 s for step time and of 0.001–0.015 m for step length, obtained from a 2D gait analysis system versus a 3D system [78]. Our results suggest that the music-cueing impacted on the participants’ gait as this was the only study which used music-cueing, but this could not be substantiated. Overall, the findings from the reliability study showed that the gait analysis system can be used with confidence to measure gait synchronisation with music beat in the main study.

### Study limitations

Due to the small sample size, the results should be considered preliminary and not generalisable to a larger population of individuals with MS. At the baseline, there was no statistically significant imbalance between groups. The female-to-male ratio indicated a probable selection bias, most likely associated with the small sample size. Thirteen females and two males were included in the study, which represents a female-to-male ratio of 6.5:1 and does not correspond to previously reported data from the UK with 2.4:1 [82] and Austria with 2.7:1 [83].

The lack of blinding is a relevant limitation of this study. To minimise this limitation, a script was used by the physiotherapist who gave the instructions and assessed participants, and support was provided consistently. Apart from the fact that blinding of the participants would not have been feasible because they would have realised their group allocation, so far, none of the interventions is known to be superior to the others. Both walking tests were performed according to internationally recognised guidelines and instructions. Nonetheless, it is possible that, even if not intentionally, the assessor might have exerted an influence through her knowledge of group allocation. Therefore, it is relevant to implement blinding of the assessors in an eventual subsequent study.

### Consequences for the main study

The results from this study suggest that a full-scale RCT is feasible. Relevant requirements elaborated for the main study are described as follows.

### Randomisation

Considering the threat of selection bias, stratified randomisation will be applied by an independent researcher to balance groups. The stratification will be according to relevant prognostic factors for a change in walking, namely, age (< 40, ≥40), gender (female, male) and disability (EDSS: 1.5–3.0, 3.5–4.5) [84]. In our previous study, we already used such a randomisation procedure on a similar population [19].

### Allocation concealment

To prevent allocation bias, allocation concealment will be performed in the main study. As with this feasibility study, each participant will be assigned a unique identification number (ID). A computer generated randomisation list based on the predefined strata will be generated by an independent researcher at the Medical University of Innsbruck. Sealed opaque envelopes including group allocation numbers 1, 2 and 3 will be created by the same researcher, which will be stored in a sequentially numbered order based on the randomisation list. These envelopes will be allocated to each participant in the order in which they are recruited. Participants will be asked to unseal the envelopes themselves and not to discuss their group allocation until study completion.

### Blinding

Involvement of three intervention groups in this feasibility study made it unlikely that the allocation to a certain group influenced participant motivation, behaviour or adherence. Compliance reports were similar in all groups, and neither the participants nor the researchers could know in advance which intervention, if any, might induce greater improvements in performance. Once they will be allocated to a treatment, participants will be aware of the type of intervention. Concerning the assessors, it is relevant to implement blinding in an eventual subsequent study.

### Assessments

During the participant selection procedure, a minimal screening of hearing will be considered since a hearing impairment could limit the participation in a (cued) MI intervention. For the evaluation of participants’ cognitive capacity and mood/depression, it seems useful to consider standardised measurement tools such as the Mini-Mental State Examination [85] and the Beck Depression Inventory [86].

### Reliability study

A further development of the reliability study will be considered for a future study, using an extended sample size and employing different assessors. Such a study could be used to confirm intra-rater reliability values and to examine test-retest reliability.

## Conclusion

Results from our feasibility study demonstrated that a full-scale RCT is feasible to investigate the mechanisms of differently cued and non-cued MI interventions and their effect on walking, fatigue and QoL in people with MS. The gait analysis instruments were found to be reliable and a future study could validate these results. Stratified randomisation using a computer-generated randomisation sequence and allocation concealment should be used in the main study. It is essential to implement blinding in a further study. Additional assessments will be considered to screen their hearing and evaluate their cognitive function and potential depression.

The preliminary improvements in walking speed, walking distance, fatigue, QoL and MI ability in the three groups are promising however need to be confirmed. After a familiarisation with cued and non-cued MI, the participants showed adequate MI ability, which could have influenced these improvements. Results from the ongoing main study shall be used to provide specific recommendations for clinically working physiotherapists on the use of (cued) MI in people with MS.

## Additional files

Additional file 1: CONSORT checklist for pilot and feasibility studies. (DOC 229 kb)

Additional file 2: Questions to be asked during phone calls. (DOCX 14 kb)

Additional file 3: Baseline characteristics for 15 participants. (XLSX 548 kb)

## Abbreviations

2D: 2-dimensional
6MWT: 6-Minute Walk Test
BPM: beats per minute
CD: compact disc
CI: confidence interval
CV MAD: coefficient of mean deviation about the median
CV: coefficient of variation
EDSS: Expanded Disability Status Scale
ICC: intra-class correlation coefficient
KVIQ-G-10: Kinaesthetic and Visual Imagery Questionnaire, German version, short form
KVIQ-G-10: Kinaesthetic and Visual Imagery Questionnaire, short form
MDC_95_: minimum detectable change with a 95% confidence interval
MFIS: Modified Fatigue Impact Scale
MS: multiple sclerosis
MSIS-29: Multiple Sclerosis Impact Scale-29
SD: standard deviation
SDC: smallest detectable change
SEM: standard error of measurement
T25FW: Timed 25-Foot Walk
TDMI: Time-Dependent Motor Imagery screening test
