# Supplementary material for: Exploring cued and non-cued motor imagery interventions in people with multiple sclerosis: a randomised feasibility trial and reliability study
Source: Arch Physiother. 2018 Mar 2;8:6. doi: 10.1186/s40945-018-0045-0 (PMC5833073; doi:10.1186/s40945-018-0045-0)
Supplement: Supplementary file 2 — Questions to be asked during phone calls. (DOCX 14 kb) [file 40945_2018_45_MOESM2_ESM.docx]

**Additional file 2**

**Questions to be asked during phone calls**

1. How has your health been since our last telephone contact and how is it at present?
2. On what days did you perform the (cued) motor imagery?
3. For how many minutes did you practise the (cued) motor imagery?
4. How is your experience of the (cued) motor imagery practise?
5. How is your experience of the motor imagery practice? (in all groups)
6. How is your experience of the music cueing? (in the MVMI and MMI groups)
7. How is your experience of the verbal cueing? (in the MVMI group)
8. Are you having any problems during or after the practice?
9. If so: what problems are you having?
10. Are you experiencing any adverse events during or after the (cued) motor imagery practice?
